# Age-Sensitive Design of Online Health Information: Comparative Usability Study

**DOI:** 10.2196/jmir.1220

**Published:** 2009-11-16

**Authors:** Richard Pak, Margaux M Price, Jason Thatcher

**Affiliations:** ^2^Department of ManagementClemson UniversityClemsonSCUSA; ^1^Department of PsychologyClemson UniversityClemsonSCUSA

**Keywords:** Internet, information organization, aging, health-related websites

## Abstract

**Background:**

Older adults’ health maintenance may be enhanced by having access to online health information. However, usability issues may prevent older adults from easily accessing such information. Prior research has shown that aging is associated with a unique pattern of cognitive changes, and knowledge of these changes may be used in the design of health websites for older adults.

**Objective:**

The goal of the current study was to examine whether older adults use of a health information website was affected by an alternative information architecture and access interface (hierarchical versus tag-based).

**Methods:**

Fifty younger adults (aged 18-23) and 50 older adults (aged 60-80) navigated a health information website, which was organized hierarchically or used tags/keywords, to find answers to health-related questions while their performance was tracked. We hypothesized that older adults would perform better in the tag-based health information website because it placed greater demands on abilities that remain intact with aging (verbal ability and vocabulary).

**Results:**

The pattern of age-related differences in computer use was consistent with prior research with older adults. We found that older adults had been using computers for less time (*F*_1,98_= 10.6, *P*= .002) and used them less often (*F*_1,98_= 11.3, *P*= .001) than younger adults. Also consistent with the cognitive aging literature, younger adults had greater spatial visualization and orientation abilities (*F*_1,98_= 34.6, *P*< .001 and *F*_1,98_= 6.8, *P*= .01) and a larger memory span (*F*_1,98_= 5.7, *P*= .02) than older adults, but older adults had greater vocabulary (*F*_1,98_= 11.4, *P*= .001). Older adults also took significantly more medications than younger adults (*F*_1,98_= 57.7, *P*< .001). In the information search task, older adults performed worse than younger adults (*F*_1,96_= 18.0, *P*< .001). However, there was a significant age × condition interaction indicating that while younger adults outperformed older adults in the hierarchical condition (*F*_1,96_= 25.2, *P*< .001), there were no significant age-related differences in the tag-based condition, indicating that older adults performed as well as younger adults in this condition.

**Conclusions:**

Access to online health information is increasing in popularity and can lead to a more informed health consumer. However, usability barriers may differentially affect older adults. The results of the current study suggest that the design of health information websites that take into account age-related changes in cognition can enhance older adults’ access to such information.

## Introduction

According to a recent Pew report on Internet usage, up to 80% of American Internet users have accessed health information on the Internet, with 64% of Americans searching for information about a specific disease and 51% searching for treatments [1]. The ease with which health consumers can access high-quality, doctor-reviewed medical information has the potential to allow patients to take more control of their health outcomes. The same Pew report shows mostly positive perceived outcomes with this ease of information access, such as feeling relieved or comforted by the information found (56%) and, more importantly, feeling confident about raising questions to the doctor after searching the Internet (56%).

However, the wealth of available information may be a curse to some health consumers: 25% of consumers who have searched for health information on the Internet felt overwhelmed by the amount of information available [1]. In addition, some sources of online health information may be of higher quality than others [2]. The issue of quality and reliability of information is problematic for interpretation when combined with health consumers’ potential lack of knowledge on health topics. Despite these downsides, access to information does seem to lead to better-perceived outcomes, such as increased confidence in patients’ perception of their health care decision-making ability [1].

### Abilities, Aging, and Internet Use

The current study examined older adults’ ability to easily access online health information. Specifically, we were interested in understanding whether interface design influenced successful access and usage of online health information sources by older adults (those age 60 and over). As a group, older adults are less active users of the Internet and Web services when compared to other age groups [3]. In addition, older adults are also more likely to suffer from various health conditions [4] and take more medications [5]. Access to quality health information might be especially beneficial to older adults’ maintenance of their more complicated health situations [6]. Being better informed may allow them to ask more questions of their health care providers or to alleviate their concerns due to lack of information.

There are a myriad of reasons why older adults are less active users of the Web. One reason is that getting older is associated with cognitive changes that make using the Web and computers in general more challenging [7-9]. Using the Internet, and more specifically navigating online information sources, places particularly heavy demands on “fluid abilities” [9]. Fluid cognitive abilities, for example working memory (the ability to hold contents in memory while attending to other things) and spatial abilities (creating and manipulating mental representations such as maps), allow us to think and act in situations that are novel[9]. Fluid abilities can be described as the means or process that allows us to learn and adapt in novel situations. When we browse or navigate a website, our performance depends on our ability to keep track of where we were in the system (working memory) and our ability to create abstract maps or models of the system (spatial abilities) [10]. As these abilities decline with age, performance on tasks that depend heavily on these abilities suffers.

However, increasing age is also associated with an increase in “crystallized intelligence” [11]. Crystallized intelligence is the nonspecific, accumulated knowledge that one gains from a lifetime of education and experience. It is the product of formal education and life experience. It is commonly measured in the laboratory with tests of vocabulary or general knowledge. In a prior study, Pak and Price [12] examined age-related changes in cognition and designed a Web interface that was adjusted for older users’ cognitive abilities. When websites were designed around keywords, or tags, instead of in a hierarchy (or folders), older users were more efficient at finding information online. In that study, younger and older users browsed a fictional travel information website to answer a series of specific questions (eg, “Where do you mail your passport application?”). Pak and Price theorized that the older adults’ advantage was due in part to their greater facility with general vocabulary and verbal ability and the interface’s reduced demands on age-sensitive spatial abilities. In their analysis, the tag-based interface placed greater demands on knowledge of vocabulary, which is an ability that grows with age; that is, the cognitive requirements of navigating in a tag-based system (compared to navigating a hierarchy) seemed to have been especially dependent on good verbal and vocabulary abilities. In many studies, including our prior study, older adults routinely outperformed younger adults on tests of vocabulary and verbal knowledge [7].

### Overview of the Study

In Pak and Price’s study [12], the travel domain was chosen to equate the amount of knowledge between younger and older adults. Prior research had shown that younger and older users did not differ in their knowledge and experience with such information [13]. However, the findings from our prior study needed replication, especially in a domain that may be more applicable to older adults: online health information. In the current study, we examined whether older adults’ navigation of a health information website would improve in a tag-based interface. There currently exists a large body of literature related to aging and usability issues on the Web (see [14] for an extensive review). However, there is far too little work on translation of this basic research into design and testing of design recommendations to improve Web usability for older adults.

## Methods

### Participants

The younger adults recruited for the study were college students, while the older adults were community-dwelling, independent-living adults recruited through newspaper advertisements. Younger adults participated for course credit or US$7/hour, whereas the older adults participated in exchange for US$7/hour.

Participants were asked about their computer experience, including length of computer experience and frequency of use. To indirectly measure experience or exposure to health-related information, participants also reported the amount of prescription medications they were taking at the time of the study. We also had participants fill out a more direct measure of health literacy, the Short Test of Functional Health Literacy in Adults (STOFHLA [15]). Finally, several ability measures were included to compare our sample of participants to typical samples used in age studies. They were a measure of general vocabulary knowledge (Shipley vocabulary test [16]), a measure of working memory (reverse digit span [17]), two measures of spatial ability (paper folding and cube comparison [18]), and finally a measure a perceptual speed (digit symbol substitution [19]).

### Web Interfaces

To create the interfaces, we first copied content from various health-related websites. The majority of information was taken from the National Institutes of Health website NIHSeniorHealth [20]. A total of 122 Web pages were captured, and the pages’ appearance was standardized (eg, same font, size, colors). These Web pages were organized using one of two information architectural schemes: tag/keyword-based or hierarchical (folders). To create these information architectures, we first grouped the 122 pages into a hierarchical system. This was done by having undergraduate students carry out a card-sorting procedure. In the procedure, students placed each page into an organizational hierarchy that made sense to them. Afterwards, they named these groupings. The results of several sessions of card sorting were merged to create the hierarchical condition that consisted of 10 top-level categories (bone and joint, cardiovascular diseases, depression, diabetes, dry mouth, hearing and vision, lung diseases, medications, skin cancer, and talking to your doctor). This same methodology was used by Pak and Price [12]. The tag-based system was created directly from the hierarchy. For example, if a page on gout treatment was organized in the hierarchical condition as Bone & Joint > Arthritis > Gout > Treatment, it was assigned the keywords “bone & joint,” “arthritis,” “gout,” and “treatment.” Our rationale for such a label assignment system was our overriding concern to keep the label names as constant as possible across both conditions. We did not want to inadvertently present more or better information in one condition over another.

**Figure 1:**
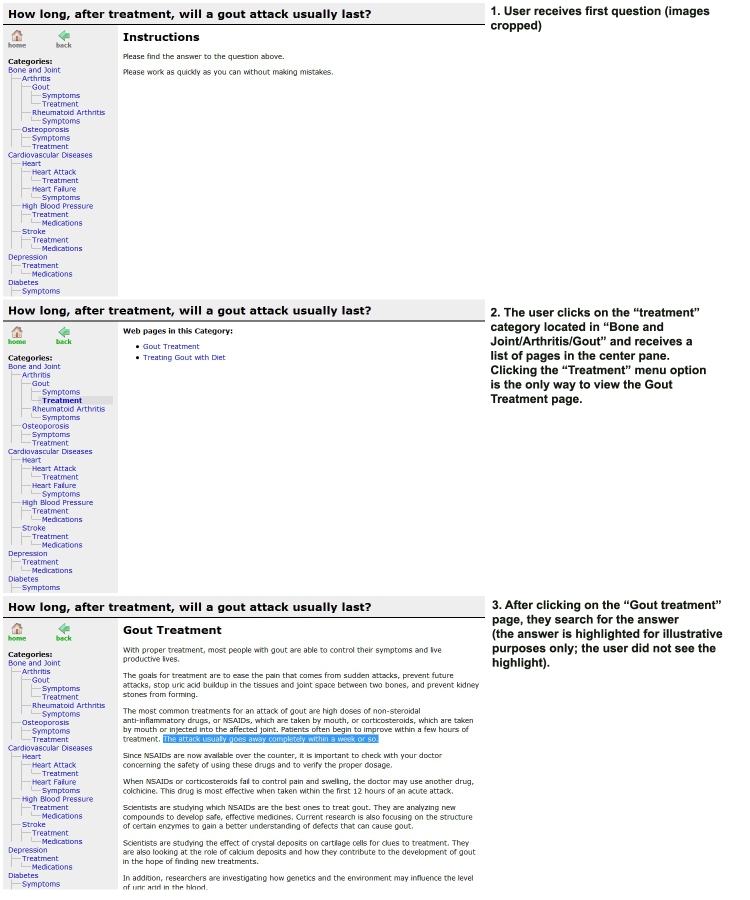
Task flow for a user in the hierarchical interface condition

**Figure 2:**
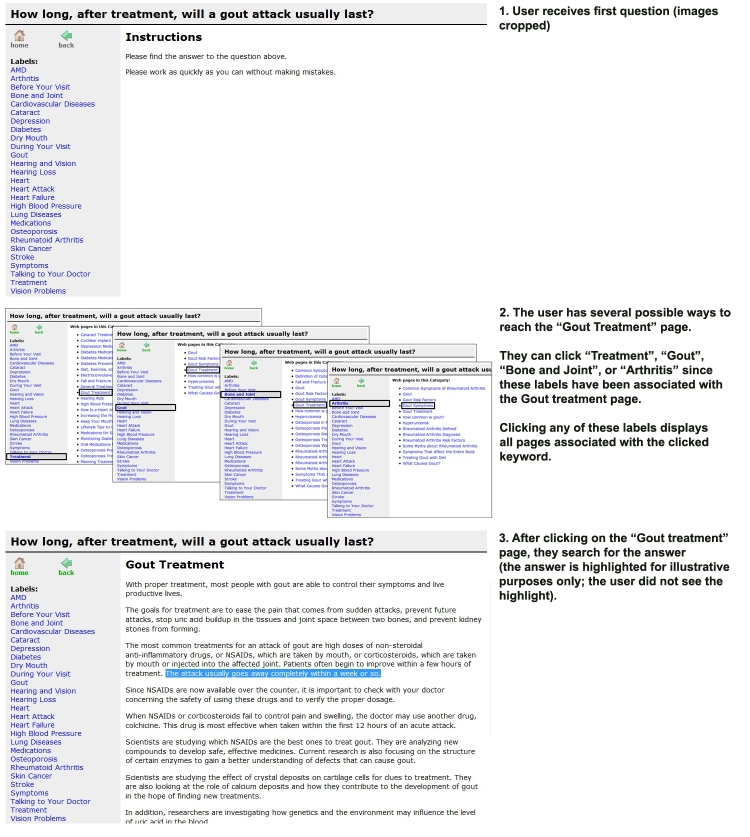
Task flow for a user in the tag-based interface condition

The main difference between the two conditions (aside from the visual difference; the hierarchical condition was visually longer) was that Web pages were only accessible in the hierarchical condition if the participant reached the single category in which it resided (ie, they had to reach the exact “folder” or “subfolder” that held the desired page). However, in the tag-based condition, Web pages could be accessed by selecting any label that was associated with the page. Figure 1 and Figure 2 show the access interfaces for the hierarchical and tag-based conditions, respectively. They illustrate the task flow for a user answering a question about gout. In both conditions, users were presented with the question at the top of the screen, the navigation interface (hierarchical or tag-based) on the left side, and the information pane on the right side.

Hierarchical organization is typically how information is organized on the Web (eg, NIHSeniorHealth). A sample hierarchical organization in our example would be the pages related to “gout” organized within the “arthritis” folder, which is within the “bones & joint” folder. The hierarchical organization is identical to how one might organize files on a computer using nested folders.

In the tag-based organizational scheme, Web pages were each labeled with keywords and the interface presented these keywords to the user. For example, if the user clicked on the gout keyword, all pages that were pre-assigned that keyword appeared (eg, a page on gout prevention, definition of gout). This is similar to how photographs are organized on the photo-sharing website Flickr.com. However, the important difference was that in our website the tags were pre-assigned by the experimenters, while on other websites tags are user generated.

### Experimental Task

Participants were asked to answer health-related questions by searching the health information site that we provided. An example question was, “What is considered normal blood pressure?” Half of the participants searched the hierarchically organized site, while the other half searched the tag-based site. When they found the answer on a specific Web page, they clicked the answer text on the page and the application presented feedback as to the correctness of the answer. A total of 25 questions were presented. The Web application was programmed in the PHP scripting language and ran on a local Web server. The application recorded the name of each visited Web page, how much time was spent on each page, and the number of times participants clicked the back button.

### Study Design and Procedure

The study was a 2 (age group: young, old) × 2 (organizational scheme: hierarchical or tag-based) factorial with age group as a grouping variable and organizational scheme as a between-group variable. Sessions were assigned to each condition and tested in a computer lab in groups of three to four people so that all participants in a session were assigned to either a hierarchical condition or a tag-based condition. The dependent variables were task completion time, errors, and mouse clicks.

Participants first filled out paperwork (eg, consent form, demographics, abilities tests) and then moved to the computer to start the information search task. Participants as a group were first guided by the experimenter through two example questions. During the example questions, participants got acquainted with the interface, and any questions were answered. After this practice session, participants were instructed to complete the search tasks as quickly but as accurately as possible and were left to complete the task on their own. These instructions were reiterated by the computer after every trial.

### Statistical Analyses

The three dependent measures of performance (task time, clicks, and errors) were subjected to a multivariate analysis of variance (MANOVA) with condition and age group as between-subject factors. In addition to an analysis of the individual dependent measures, a composite variable with the three dependent measures was created. The benefits of creating a composite are increased stability of measurement. A composite performance variable incorporating task completion time, error rate, and number of steps was created for analysis. Each dependent variable was normalized (*z*score transformed), and these individual *z*scores were averaged to create a unit-less composite performance variable that ranged from 1 (worst performance) to −1 (best performance). The analysis of composite variables in usability evaluation has been suggested by researchers as a way to increase stability of measurement [19] and ease interpretation [19,21]. This composite performance measure was subjected to a 2 × 2 analysis of variance (ANOVA).

## Results

### Participant Characteristics

Fifty younger adults (29 female) ranging in age from 18 to 23 (mean = 19.5, SD = 1.7) and 50 older adults (27 female) ranging in age from 60 to 80 (mean = 70.6, SD = 5.6) participated in the study.

There were significant age group differences in total length of computer experience and frequency of use, with younger adults using computers for a longer period of time than older adults (*F*_1,98_= 10.6, *P =* .002, *η*_*p*_^2^= .10) and more frequently (*F*_1,96_= 11.3, *P =* .001, *η*_*p*_^2^= .1). Older adults reported taking significantly more prescriptions than younger adults (*F*_1,98_= 57.7, *P <* .001, *η*_*p*_^2^= .37). According to the STOFHLA, there were no age group differences in health literacy. Older adults typically outperform younger adults on tests of vocabulary, and this was the case in our sample, with the older adults outperforming the younger adults (*F*_1, 98_= 11.4, *P*= .001, *η*_*p*_^2^= .11). Younger adults, however, outperformed the older adults in the fluid ability measures (memory span *F*_1,98_= 5.7, *P*= .02, *η*_*p*_^2^= .06; spatial visualization *F*_1,98_= 34.6, *P*< .001, *η*_*p*_^2^= .27; spatial orientation *F*_1,98_= 6.8, *P*= .01, *η*_*p*_^2^= .07; and perceptual speed *F*_1,98_= 70.2, *P*< .001, *η*_*p*_^2^= .42), which is consistent with the general literature on aging and cognition [7].

General participant characteristics are presented in Table 1.

**Table 1:** Younger and older user characteristics by condition

	Age Group *P*^a^	Younger Users					Older Users				
		Hierarchical		Tag-Based		*t*^b^	Hierarchical		Tag-Based		*t*^b^
		Mean	SD	Mean	SD		Mean	SD	Mean	SD	
Age (years)		19.4	1.7	19.6	1.8	0.4	70.6	5.8	69.4	5.5	−0.8
Length of computer use^c^	.002	5.0	0.0	5.0	0.2	−1.0	4.5	1.2	4.5	0.9	0.1
Frequency of computer use^d^	.001	5.9	1.0	5.6	1.0	−1.1	5.0	1.4	4.8	1.6	−0.5
Number of medications taken^e^	< .001	0.9	1.2	1.0	1.4	0.3	4.1	2.8	5.2	3.5	1.2
Health literacy^f^	.12	35.2	1.1	35.5	0.7	1.2	34.6	2.6	35.1	1.3	0.9
Vocabulary^g^	.001	30.2	3.2	27.2	5.9	−2.2	37.6	20.4	34.6	4.6	−0.7
Memory span^h^	.02	10.6	2.0	9.8	2.9	−1.1	8.3	3.0	9.5	2.6	1.5
Spatial visualization^i^	< .001	5.8	2.1	6.0	2.0	0.4	3.5	1.6	4.2	1.4	1.6
Spatial orientation^j^	.01	9.8	3.2	10.9	4.0	1.1	8.6	2.8	8.8	2.5	0.2
Perceptual speed^k^	< .000	66.7	10.3	66.0	9.9	−0.3	49.1	10.8	50.3	8.6	0.4

^a^One-way ANOVA.

^b^ *t*tests showed no significant condition differences (within each age group) at *P* *<*.001 (stricter *P*criterion used to compensate for inflated degrees of freedom due to multiple comparisons).

^c^ Total length of computer experience on a scale of 1 (less than 6 months) to 5 (greater than 5 years).

^d^ Frequency of computer use on a scale of 1 (once every few months) to 7 (daily, most of the day).

^e^ Prescription medications only.

^f^ Test of health literacy composite score (STOFHLA) [15]; higher equals better health literacy.

^g^ Shipley vocabulary score; higher is better [16].

^h^ Reverse digit span [17].

^i^ Paper folding test [18].

^j^ Cube comparison test [18].

^k^Digit symbol substitution (number correct [19]).

### Performance

**Figure 3:**
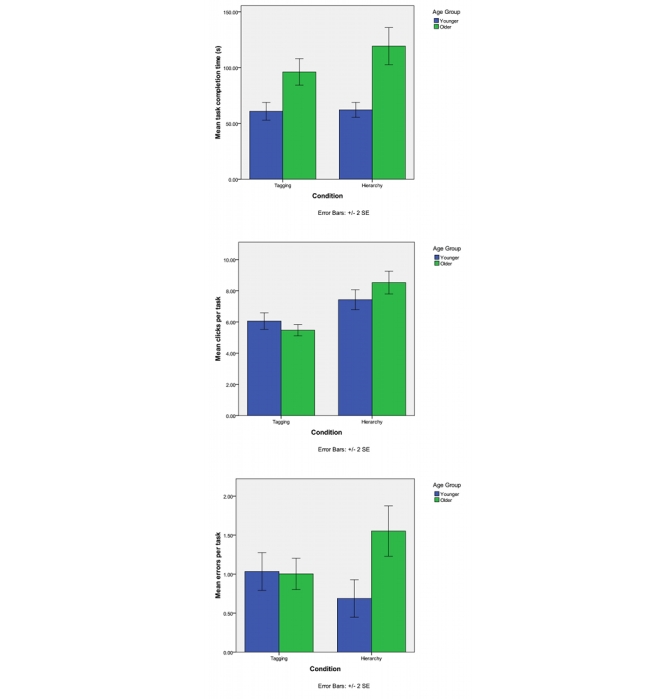
Time, errors, and clicks as a function of condition and age group (error bars represent standard error)

Performance (mean task completion times, mean number of mouse clicks to completion, and mean errors per task) is shown in Table 2 and illustrated in Figure 3.

**Table 2:** Measures of performance as a function of condition and age group

		Younger Users		Older Users	
		Mean	SD	Mean	SD
Mean task completion time (s)					
	Tag-based	60.90	19.86	96.17	29.70
	Hierarchy	62.16	16.78	119.31	41.62
Mean clicks per task					
	Tag-based	6.06	1.32	5.47	.91
	Hierarchy	7.43	1.60	8.52	1.83
Mean errors per task					
	Tag-based	1.00	.50	1.02	.55
	Hierarchy	1.55	.81	1.12	.83

The results of the analysis of dependent measures showed significant overall main effects of condition and age group (*F*_1,94_= 23.8,*P*< .001, *η*_*p*_^2^= .43, *F*_1,94_= 29.5, *P*< .001, *η*_*p*_^*2*^= .48, respectively), and the interaction was significant (*F*_1,94_= 4.7, *P*< .001, *η*_*p*_^2^= .13), so the main effects were not followed up. Follow-up analysis revealed that the significant interaction was due to a significant interaction in mean clicks (*F*_1,96_= 8.4, *P*< .001, *η*_*p*_^2^= .04) and mean errors (*F*_1,96_= 12.29, *P*< .001, *η*_*p*_^2^= .11). The interaction in mean task time was not significant (*P*= .06). The source of the interaction in mean clicks and errors was not significant age difference in clicks or errors in the tag condition but in the taxonomy (hierarchical) condition: older adults made more clicks than younger adults. These interactions are illustrated in Figure 3. These results conceptually replicate the earlier results [12] that showed that navigation through a website can be improved if the organization is organized around keywords, not a hierarchy.

**Figure 4:**
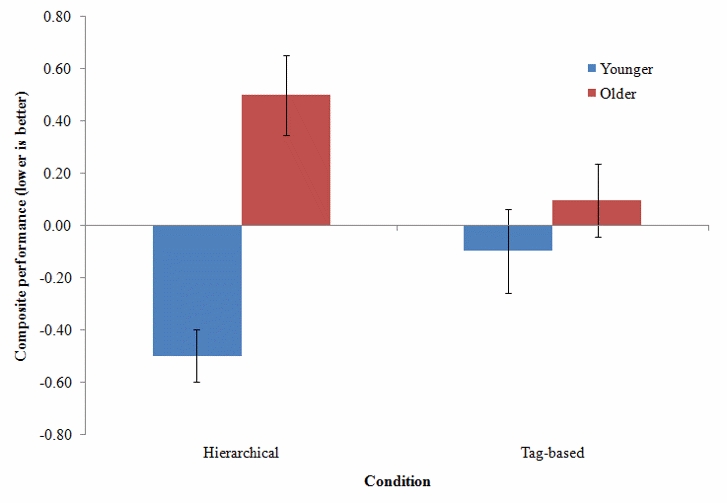
Performance as a function of condition and age group (error bars represent standard error)

The analysis of the composite measure of performance showed that the main effect of age group was significant (*F*_1,96_= 18.0, *P* <.001, *η*_*p*_^2^= .16), indicating that older adults in general performed worse than younger adults. There was no significant main effect of condition; however, the age group × condition interaction was significant (*F*_1,96_= 8.1, *P =* .005, *η*_*p*_^2^= .08), indicating that condition (hierarchical vs tag-based) differentially affected each age group. Post hoc analyses (Bonferonni method) revealed that the source of this interaction was the hierarchical condition. Younger adults significantly outperformed older adults in the information search task when using the hierarchical condition (*F*_1,96_= 25.2, *P <*.001, *η*_*p*_^2^= .21). However, in the tag-based condition, there were no significant performance differences between the younger and older adults in the information search task. This replicates our earlier findings that a tag-based system that relies on the generation and recognition of keywords may benefit older adults’ information-finding performance. The interaction is represented in Figure 4.

The current finding that older users’ performance searching for medical information through a tag-based system was better than through a hierarchically organized system replicates our earlier findings examining travel information. However, we also wanted to examine how pre-existing domain knowledge or experience with health and medical information might be related to performance in each of the two organizational schemes. Table 3 shows the correlations between performance, number of medications, and experience measures (computer experience and health/medical experience).

In the hierarchically organized condition, performance was significantly correlated with age (older adults performed worse than younger adults), the number of medications currently taken (increased number of medications was associated with worse performance), and health literacy (those with greater health literacy performed better). It is somewhat intuitive that health literacy (STOFHLA) was associated with better performance in the information search task, but it is puzzling that performance was not correlated with number of medications taken. We initially assumed that number of medications could be used as an indirect indicator of health knowledge, with the assumption that people who took more medications would have a greater amount of health knowledge because of their need to manage and understand their medication regimen. However, the correlation between STOFHLA and number of medications was not significant. One possibility could be that number of medications taken is a better indicator of overall cognitive and physical health status (with those taking more medications having worse health and thus cognitive status), and not knowledge.

**Table 3:** Correlations between health literacy and knowledge and performance in hierarchical and tag-based conditions

Hierarchical Condition					
		1	2	3	4
1	Age	-			
2	Number of medications	**0.6**	-		
3	STOFHLA^a^	−0.2	0.0	-	
4	Composite performance^b^	**0.6**	**0.3**	−**0.5**	-
Tag-Based Condition					
		1	2	3	4
1	Age	-			
2	Number of medications	**0.6**	-		
3	STOFHLA^a^	−0.2	−0.1	-	
4	Composite performance^b^	0.2	−0.1	−0.1	-

^a^ Health literacy; higher is better.

^b^ Composite performance was reverse coded (lower is better).

^c^Boldface indicates significant correlations at *P*< .05.

In contrast, the pattern of correlations in the tag-based system show that performance was not significantly associated with age, number of medications, or health knowledge. These results are consistent with those of Pak and Price [12], which showed that age was not a significant predictor of performance in the tag-based condition but did predict significant variance in the hierarchical condition. The implication is that hierarchical systems are sensitive to age (and thus age-related differences in abilities and knowledge), while tag-based systems are not.

## Discussion

The online health information consumer ranges widely in age, experience with computers, and health status [1,22]. It is thus critical to examine usability issues that assure people with varying backgrounds can successfully access online health information. The goal of the current study was to determine whether older adults’ health information search and retrieval performance could be improved with relatively modest interface usability changes based on the cognitive aging literature. Earlier work showed that when older adults searched through a tag-based website, their performance was relatively better than when they searched through a hierarchically organized website. The basis for improvement was theorized to be that tag-based interfaces, compared to hierarchical organizations, shift cognitive demands from spatial abilities (ie, knowing where you are) to verbal/vocabulary abilities (knowing keywords).

The results from the current study show that with a restructuring of the information architecture and the access interface (from a hierarchical organization to one based on keywords or tags), older adults are able to improve their information search and retrieval performance. This was presumably the result of a change in the ability demands of each system, with the tag-based system placing less stringent demands on older adults’ cognition than the hierarchical system. This study represents the first replication of our earlier results, but with a topical domain that may be especially relevant to older adults: online health information.

While older adults’ performance was improved in a tag-based interface, younger adults’ performance was worse in the tag-based system. This may be due to the simultaneous reduced spatial ability demands and increased verbal/knowledge demands from the tag-based interface coupled with younger adults’ relative lack of verbal abilities (compared to older adults). This suggests the possibility that, for optimal performance, online health information providers may need to provide age-specific interfaces for their users. However, it also stresses the need for further research into interfaces that can combine the beneficial aspects of tag-based and hierarchical interfaces useful for people of all ages. One such novel interface, faceted navigation, is being used on some websites and may be the bridge between hierarchical interfaces and pure tag-based interfaces [23]. In facetted navigation systems, users progressively narrow search results by selecting facets, or dimensions, of information. For example, a free-text search of diabetes from a health information site might return several thousand results. In a facetted navigation system, the user could then focus on facets of those results (eg, only news articles, or peer-reviewed research) by selecting keywords. The user experience is similar to tag-based interfaces, but the difference is that the presentation of tags can be hierarchical.

### Limitations

One limitation of this research is that online medical information websites or portals may only be one of several possible destinations for health consumers [1,24]. For example, information searchers may turn to generic search engines (eg, Google, Yahoo), which brings unique usability problems [24,25]. In a usability study of Web-based health information, Eysenbach and Köhler found that older users exhibited suboptimal search engine use such as viewing only the initial search results page (when more were available) [24]. Another limitation is related to the design of the materials. Because of our desire to keep the hierarchy and tag-based conditions as conceptually consistent as possible, the hierarchy (presented on the left-hand side) was slightly visually taller than the tag-based condition. However, we believe that this difference was minor and is unlikely to, by itself, explain the observed effects. For example, it could be the case that the longer hierarchy (and concomitant increase in visual clutter) may explain older adults performing more poorly in the hierarchical condition compared to the tag-based condition. However, the clutter explanation does not explain why younger adults performed relatively better in the presence of the hierarchical condition compared to tag-based—they would also be subject to clutter effects.

### Conclusions

The current research was an attempt to add evidence-based knowledge to the problem of older adult information searching on the Web. There is now nearly a critical mass of literature on age-related changes as they may relate to the use of Web resources [14], but much work is necessary to translate this basic knowledge of age differences into specific design recommendations and recommendations for older adults.

## Abbreviations

STOFHLA: Short Test of Functional Health Literacy in Adults
